# Discovery of genomic variations by whole-genome resequencing of the North American Araucana chicken

**DOI:** 10.1371/journal.pone.0225834

**Published:** 2019-12-10

**Authors:** Rooksana E. Noorai, Vijay Shankar, Nowlan H. Freese, Christopher M. Gregorski, Susan C. Chapman

**Affiliations:** 1 Clemson University Genomics and Bioinformatics Facility, Clemson University, Clemson, South Carolina, United States of America; 2 Center for Human Genetics, Clemson University, Greenwood, South Carolina, United States of America; 3 Department of Bioinformatics and Genomics, University of North Carolina at Charlotte, Charlotte, North Carolina, United States of America; 4 Department of Biological Sciences, College of Science, Clemson University, Clemson, South Carolina, United States of America; Xiamen University, CHINA

## Abstract

*Gallus gallus* (chicken) is phenotypically diverse, with over 60 recognized breeds, among the myriad species within the Aves lineage. Domestic chickens have been under artificial selection by humans for thousands of years for agricultural purposes. The North American Araucana (NAA) breed arose as a cross between the Chilean “Collonocas” that laid blue eggs and was rumpless and the “Quetros” that had unusual tufts but with tail. NAAs were introduced from South America in the 1940s and have been kept as show birds by enthusiasts since then due to several distinctive traits: laying eggs with blue eggshells, characteristic ear-tufts, a pea comb, and rumplessness. The population has maintained variants for clean-faced and tufted, as well as tailed and rumplessness traits making it advantageous for genetic studies. Genome resequencing of six NAA chickens with a mixture of these traits was done to 71-fold coverage using Illumina HiSeq 2000 paired-end reads. Trimmed and concordant reads were mapped to the Gallus_gallus-5.0 reference genome (galGal5), generated from a female Red Junglefowl (UCD001). To identify candidate genes that are associated with traits of the NAA, their genome was compared with the Korean Araucana, Korean Domestic and White Leghorn breeds. Genomic regions with significantly reduced levels of heterogeneity were detected on five different chromosomes in NAA. The sequence data generated confirm the identity of variants responsible for the blue eggshells, pea comb, and rumplessness traits of NAA and propose one for ear-tufts.

## Introduction

**Fig 1:**
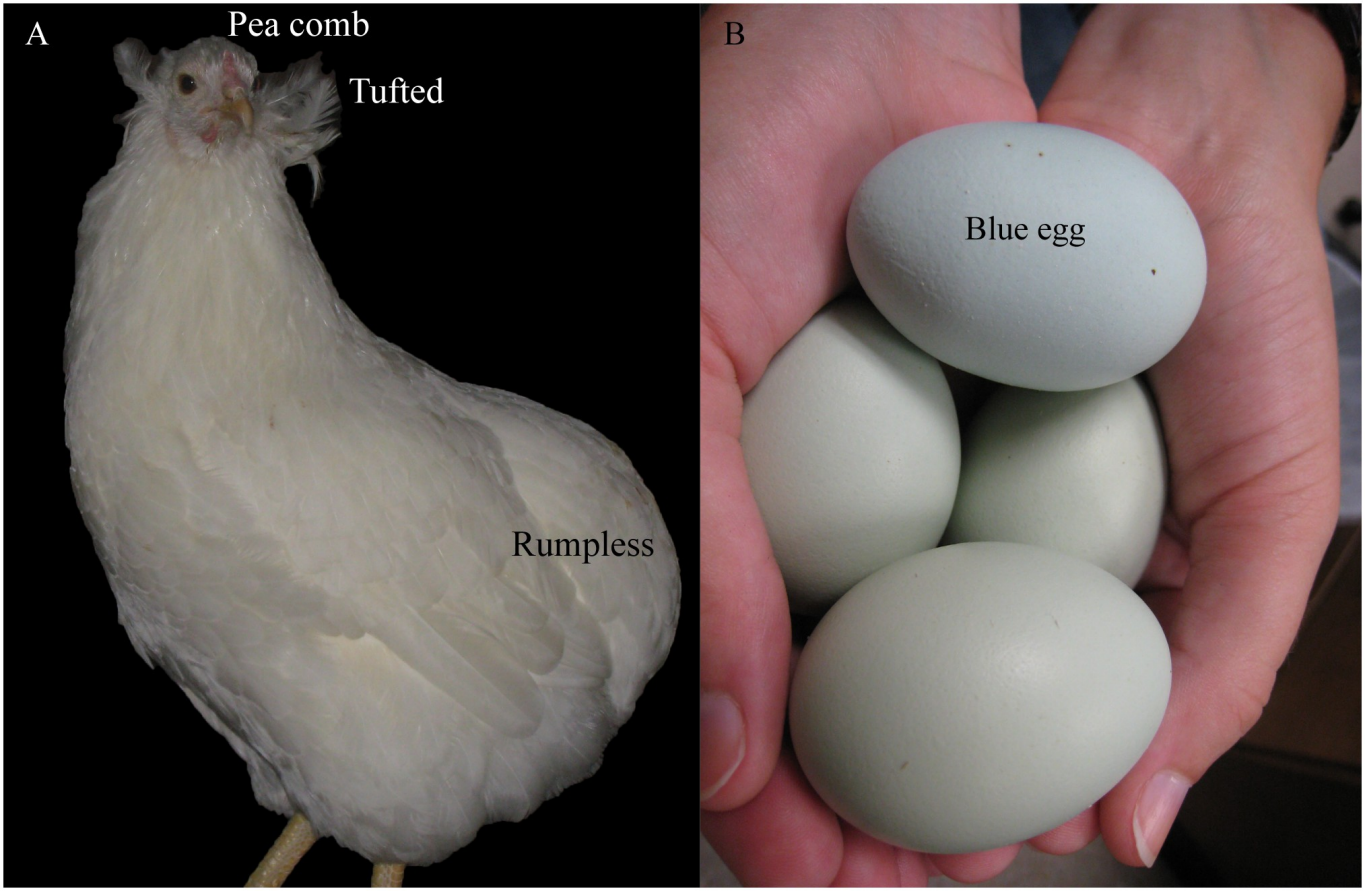
Photographs of the four traits known to be associated with North American Araucanas (NAAs). (A) NAA with ear-tufts, a pea comb, and rumplessness and (B) NAA laid blue shelled eggs.

The Red Junglefowl is considered ancestral to the domestic chicken (*Gallus gallus domesticus*) [1]. Through natural and human selection over 60 breeding lines are now recognized by the American Poultry Association [2], and these breeds incorporate 211 different phenotypes [3], making it diverse among the myriad species within the Aves lineage. Selected traits include morphological features such as body size (bantam, broiler), plumage, egg-laying ability and egg color (white, brown, blue), and comb size and shape. As a result, chickens within the *Gallus* subspecies are phenotypically diverse, among the hugely diverse Galliform order that contains 290 species. Chickens are of worldwide agricultural importance due to their meat and eggs being consumed as food [4]. In addition, chickens are also useful for the study of multiple bacterial and viral diseases, and for modeling genetic diseases that affect humans, as well as a key model organism for developmental biology research [5–7]. Comparative genomics studies to dissect phenotypic variants at the sequence level are becoming prevalent due to lower genome sequencing costs. Identifying conserved and breed-specific variants, and the regulatory elements controlling these regions, is important in understanding genome architecture, and how unique, breed-specific characteristics arise [8,9]. Commercial inbred lines such as the White Leghorn (WL) have reduced genetic diversity, whereas lines maintained for particular traits by enthusiasts are often both incrossed and outcrossed, and are genetically more diverse. The NAA breed arose as a cross between the Chilean “Collonocas” that laid blue eggs and was rumpless, and the “Quetros” that had feather-covered, boney protrusions from the sides of their faces called tufts but was tailed. NAAs were introduced from South America in the 1940s and have been kept as show birds by enthusiasts since then due to several distinctive traits, one of which is laying eggs with blue eggshells [10,11]. Humans use artificial selection to influence whole populations of livestock and pets, including chickens, cows, and dogs [12]. The NAA is characterized by three distinct phenotypes: ear-tufts, a pea comb, and rumplessness. Several color variations are considered acceptable for the breed standard, and NAA additionally lay blue-shelled eggs, Fig 1 shows photographs of the four traits. Candidate genes for two non-linked phenotypes rumplessness and ear-tufts were reported in NAA [13]. Misexpression of *IRX1* and *IRX2* proneural genes, in a rare gain-of-function mechanism, results in disruption of the bipotential tailbud mesenchyme progenitor population, which drives cells toward a neural fate at the expense of the mesoderm lineage. This defect in axis elongation causes premature termination of caudal extension and the characteristic rumpless phenotype [14]. Here, we extend our findings through validation of two candidate causative SNPs upstream of the *IRX1/2/4* cluster.

Similarly, a heterozygous region (0.93–1.51 Mb) on chromosome 15 including *TBX1* and *GNB1L* was associated with the ear-tufted phenotype [13]. Through structural variant analysis of the same chromosome, a hemizygous deletion within the previously identified region showed association with ear-tufts in NAAs. As expected, all clean-faced birds lacked this particular deletion in that region. Furthermore, in this paper we identify several candidate genes that showed association to the NAA breed, over the other breeds in the study, and may point to additional NAA specific traits. For this reason, chicken breeds, such as the North American Araucana (NAA), are a good model organism for identification of genomic variants.

**Fig 2:**
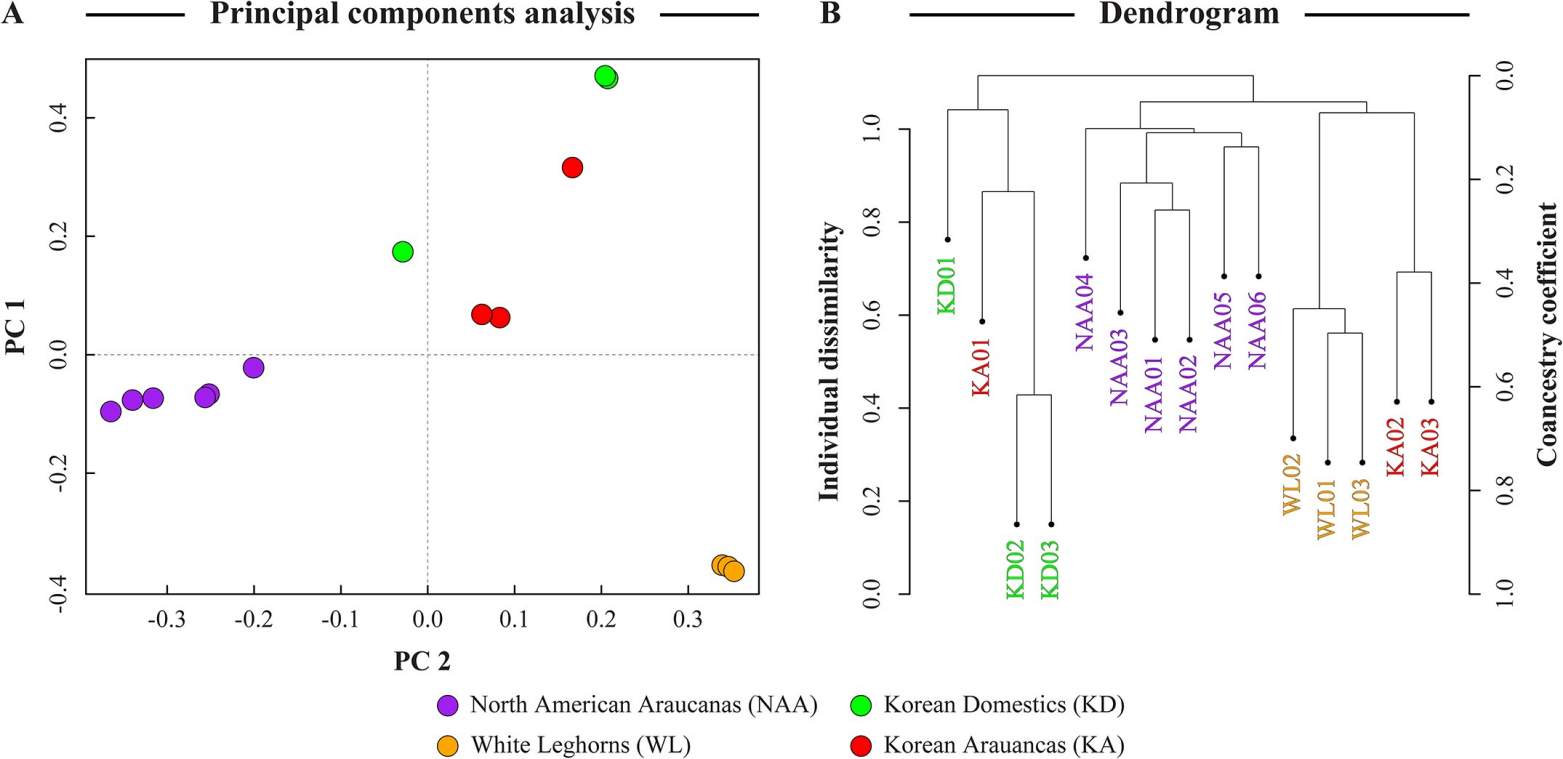
The relationship between groups of birds. (A) Principal Components Analysis and (B) Dendrogram. Colors of dots in PCA and labels in dendrogram correspond to different groups of birds. Violet dots and text represent North American Araucana, green dots and text represent Korean Domestics, gold dots and text represent White Leghorns, and red dots and text represent Korean Araucana.

To examine the genetic architecture of the NAA genome, whole-genome resequencing of six Araucana birds was performed: one tailed and five rumpless, of which four had ear-tufts and two were clean-faced (Table 1). To identify breed specific NAA traits, alignments of the NAA Illumina HiSeq 2000 paired-end reads were compared to the Gyeongbuk/Korean Araucana (KA) and Korean Native Domestic (KD) breeds, as well as the highly inbred WL commercial line [15,16]. Results of the sequence comparison between KA, KD, and WL from PRJNA291174 have been described in Fig 2A and 2B of Jeong H, et al. 2016 [15]. The relationship between these chickens seen in 2A and 2B are similar to what was found in our study. Both NAA associated SNPs and associated regions were used to identify candidate genomic characteristics related to actin binding (*TAGLN3*) and cytoskeleton (*MYH1D*, *MYH1F*), metabolism (*NAXD*), vesicle trafficking associated genes (*AP2B1*, *EXOC4*, *SEC24D*, *STX2*, *TSNARE1*, *TXLNG*), and many others. A flowchart of methods can be seen in S1 Fig.

**Table 1:** Phenotypes for NAA selected for whole-genome resequencing.

ird id	Flock Location	Sex	Color	Tufted or Clean Faced	Rumpless or Tailed
**NAA01**	S.C.^1^	M	White	Tufted	Rumpless
**NAA02**	S.C.^1^	M	White	Tufted	Rumpless
**NAA03**	S.C.^1^	U	White	Clean	Rumpless
**NAA04**	N.J.	F	Blue	Tufted	Rumpless*
**NAA05**	N.J.	M	Black Breasted Red	Clean	Tailed
**NAA06**	S.C.^2^	M	Black	Tufted	Rumpless*

^1^ S.C. location 1

^2^ S.C. location 2

*Heterozygous for rumpless

## Results

### Sequencing and variant detection

The number of raw reads produced per sample ranged from 103 to 215 million. Trimmed paired-end reads were mapped to the Gallus gallus-5.0 reference genome (galGal5) [17], generated from a female Red Junglefowl (UCD001). Genome coverage ranged from 9.2X–18.8X (median 11.39X); resulting in a combined 71.0-fold coverage for the NAA (six birds), a 39.6-fold coverage for the KA (three birds), a 35.1-fold coverage for the WL (three birds), and a 31.8-fold coverage for the KD (three birds) (S1 Table).

Putative variants were determined by comparing the aligned reads of all 15 birds with the reference sequence (galGal5). More than 17.5 million variants were identified across the genome, including unmapped linkage groups. These variants were filtered to 9,422,906 single nucleotide polymorphis (SNPs) by including only biallelic SNPs on the autosomes, with 0% missing data across all 15 birds, and the following minimum qualities: read depth ≥ 3, MAPQ > 40, and QUAL > 40. This filtered list of SNPs was used as input for the variant effect predictor (VEP) analyses. These variants were on chromosomes 1–28, 30, 32, 33, and LGE64. Chromosomes Z and W were excluded due to inclusion of an unknown number of male and female birds.

### Identifying SNPs associated with NAAs

The first method used to identify genes associated with the NAA breed was to find SNPs that were significantly discriminatory when comparing the NAA breed to the others in this study. The dataset was further reduced from ~9.42 to 9.17 million SNPs using SNPRelate, a program that uses principal components analysis (PCA) to handle population structure and identity-by-decent to determine relatedness between samples to identify informative SNPs. This program removed 252,528 SNPs using the default parameters. A PCA plot showing the definitive separation of the six NAA and the three WL from the other chickens can be seen in Fig 2A. For two out of three of the KA and two out of three of the KD there is also clear separation from each of the other three groups. The remaining KA and KD samples show a discrepancy in position between them and the other two samples in their respective group. It is possible that the two outliers were switched during sequencing or are mislabeled in the SRA database and that the three KA and three KD samples should have tightly cluster. The overall separation between groups is predominantly on the x-axis (PC 2–14.57% of the variation), implying that the second-largest gradient of variability corresponds to SNPs that separate groups. Along the Y-axis (PC1–18.06% of the variation), there is separation of the WL from the other three groups, which in turn show less separation from each other as whole groups.

The clustering patterns revealed by PCA are further delineated in the dendrogram where chickens that belong to the same group appear in clades (Fig 2B). For example, the six NAA, and the three WL samples appear in their corresponding clades. KA and KD appear in their clades, and their individual outliers (KA01 and KD01) appear outside of their clades. The KD clade and the KA01 and KD01 outlier samples are shown to be most different from the NAA, KA and WL clades.

**Fig 3:**
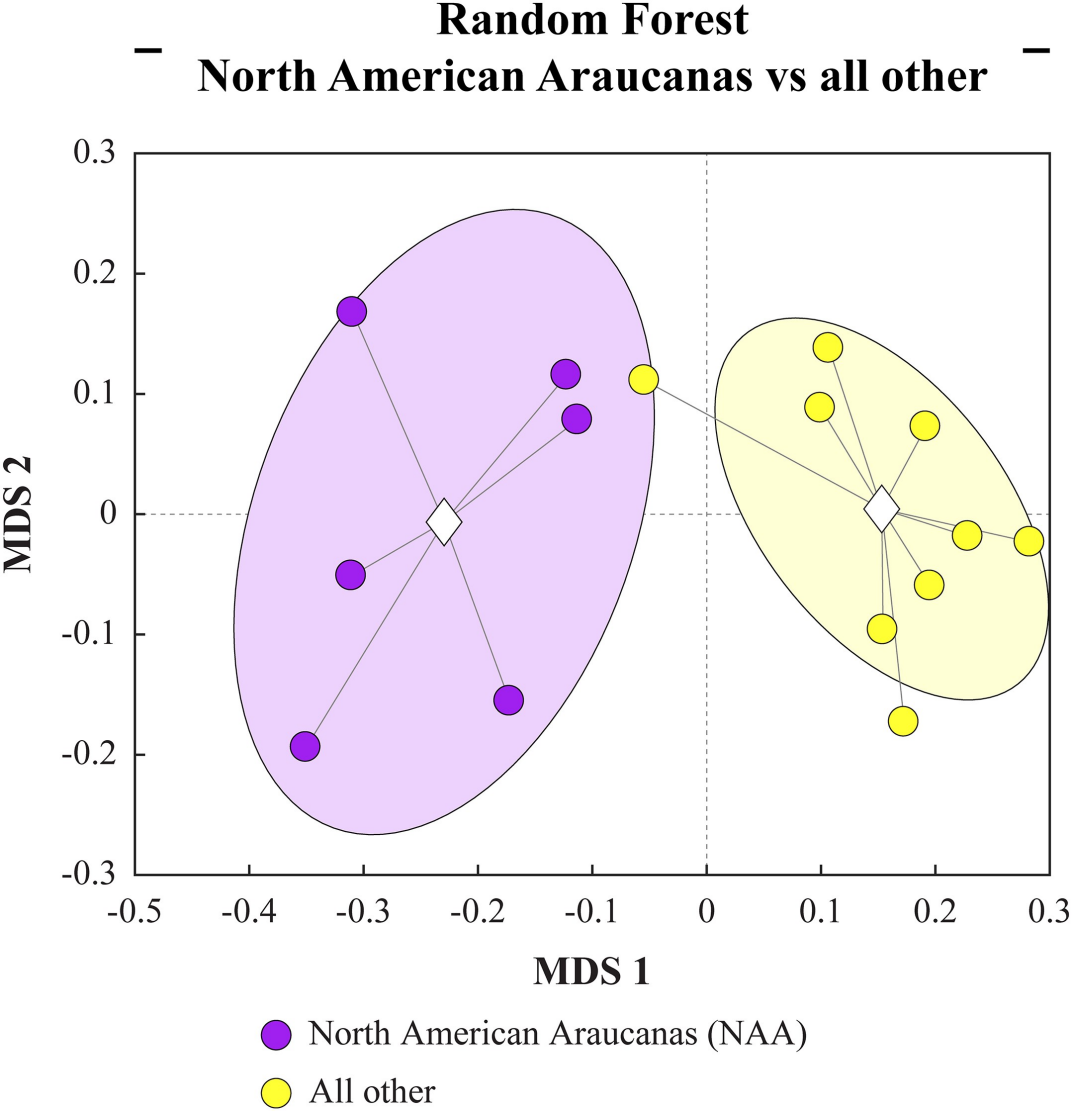
Visualization of multidimensional scaling plot. Discriminant model from random forest analysis on North American Araucanas (NAAs) compared to all other groups. The ellipses represent three standard errors around the centroid for each group. Violet dots represent NAA. Yellow dots represent the combined group of birds from Korean Domestics, White Leghorns and Korean Araucana.

To identify the SNPs that explicitly discriminate NAAs from the other three breeds of chickens the SNPs identified by SNPRelate were input into a machine learning classifier based on decision trees called random forest (RF). The goal of this algorithm is first to construct a discriminant model that separates groups based on their variable values. In addition to the construction of the discriminant model, it also ranks the variables based on their contribution to the discrimination between groups. This is done by randomizing the values of each variable, reconstructing the discriminant model and assessing the loss in discriminatory power due to the randomization of the variable. This functionality was exploited to identify the SNPs that define NAA with reference to the other three breeds (KA, KD, and WL) in the comparison. The results of this analysis identified 203 out of 9,170,378 markers as the top discriminatory SNPs (S2 Table). These markers are distributed among 21 chromosomes (1–20, 26, and 28). The validity and discriminatory performance of the RF model can be seen in the multidimensional scaling (MDS) plot of the proximity matrices (Fig 3). The p-value for cluster separation in the RF MDS plot (based on the Monte Carlo permutation procedure of the Davies-Bouldin Index) is <0.001.

Annotation of each SNP from the separation of the NAA to the other three groups with its VEP results (S2 Table) found that 77 of these markers are in intergenic regions, and 126 are within a region of a gene. Of the markers within genes, their locations are 5’ UTR variant (1), intron variants (94), upstream gene variants (13), synonymous variant (1), and downstream variants (17). Surprisingly, none of the markers were associated with amino acid changes within the coding region of the associated genes.

Twenty-six candidate genes were selected for further analyses using the top, in order of importance for classification (based on Mean Decrease in Accuracy overall: column E, S2 Table), 22 markers that are within genes and fit the pattern of the alternate allele being present at a higher frequency in the NAA samples (Table 2). Several markers were associated with more than one gene. The ENSAGALG gene names refer to novel genes in the chicken annotation.

**Table 2:** Candidate genes selected based on annotation of the top 22 markers found to be within genes and fitting the pattern of the alternate allele being present at a higher frequency in the NAA samples.

Ensembl ID	Gene name	Gene description	VEP results	snpEff results
ENSGALG00000043223	*COL26A1*	collagen type XXVI alpha 1 chain	intron variant	intron variant
ENSGALG00000011630	*GLI2*	GLI family zinc finger 2	intron variant	intron variant
ENSGALG00000041098	*novel gene*	novel gene	intron variant	intron variant
ENSGALG00000015211	*DTNA*	dystrobrevin alpha	intron variant	intron variant
ENSGALG00000014974	*MIB1*	mindbomb E3 ubiquitin protein ligase 1	intron variant	intron variant
ENSGALG00000005657	*CRHR2*	corticotropin-releasing factor receptor 2	intron variant	intron variant
ENSGALG00000046171	*novel gene*	novel gene	intron variant	NA
ENSGALG00000002242	*GALNT9*	polypeptide N-acetylgalactosaminyltransferase 9	NA	intergenic region
ENSGALG00000002272	*NOC4L*	nucleolar complex associated 4 homolog (NOC4L)	NA	intergenic region
ENSGALG00000004756	*CEP89*	centrosomal protein 89	upstream gene variant	upstream gene variant
ENSGALG00000004814	*RHPN2*	Rhophilin Rho GTPase binding protein 2	downstream gene variant	intergenic region
ENSGALG00000034534	*FAAP24*	Fanconi anemia core complex associated protein 24	downstream gene variant	downstream gene variant
ENSGALG00000006562	*MCF2*	MCF.2 cell line derived transforming sequence	intron variant	intron variant
ENSGALG00000036938	*RALYL*	RALY RNA binding protein like	intron variant	intron variant
ENSGALG00000029378	*ITGA9*	integrin alpha-9 precursor	intron variant	intron variant
ENSGALG00000000908	*ADAT1*	tRNA-specific adenosine deaminase 1	intron variant	intron variant
ENSGALG00000008477	*EXOC4*	exocyst complex component 4	intron variant	intron variant
ENSGALG00000015379	*TAGLN3*	transgelin 3	intron variant	intron variant
ENSGALG00000015307	*ABI3BP*	ABI family member 3 binding protein	intron variant	intron variant
ENSGALG00000011910	*MNAT1*	MNAT1, CDK activating kinase assembly factor	intron variant	intron variant
ENSGALG00000017389	*SIX4*	SIX homeobox 4	upstream gene variant	upstream gene variant
ENSGALG00000031741	*PTK2*	Focal adhesion kinase 1	intron variant	intron variant
ENSGALG00000042125	*AP2B1*	adaptor related protein complex 2 beta 1 subunit	intron variant	intron variant
ENSGALG00000037890	*novel gene*	novel gene	NA	upstream gene variant
ENSGALG00000016845	*NAXD*	NAD(P)HX dehydratase	intron variant	intron variant
ENSGALG00000042644	*novel gene*	novel gene	downstream gene variant	downstream gene variant
ENSGALG00000016547	*TXLNG*	taxilin gamma	upstream gene variant	upstream gene variant
ENSGALG00000016548	*SYAP1*	synapse associated protein 1	NA	intergenic region
ENSGALG00000010352	*ARHGEF26*	Rho guanine nucleotide exchange factor 26	intron variant	intron variant
ENSGALG00000041255	*ADAM12*	disintegrin and metalloproteinase domain-containing protein 12 isoform 2 precursor	intron variant	intron variant

Reannotation of each of the 22 markers with snpEff was combined with the results from VEP (Table 2). This table presents the implications of the presence of the alternate allele SNPs found to be significant in NAA, based on the criteria above. All of the markers are predicted as modifiers using VEP and/or snpEff. SnpEff added four genes to the results: *GALNT9* (polypeptide N-acetylgalactosaminyltransferase 9), *NOC4L* (nucleolar complex associated 4 homolog), ENSGALG00000037890, and *SYAP1* (synapse associated protein 1).

Since these significant markers were all non-coding variants, it can be considered that they might have regulatory functions over nearby genes. Linkage disequilibrium breaks down at about 40kb in chickens [18]. To this end, all genes within 40kb on either side of each marker were identified and listed in S3 Table. Due to increasing the distance from the SNP to these genes, further work would need to be done to validate any regulatory effects, and that is beyond the scope of this work.

### Identifying regions associated with NAAs

**Fig 4:**
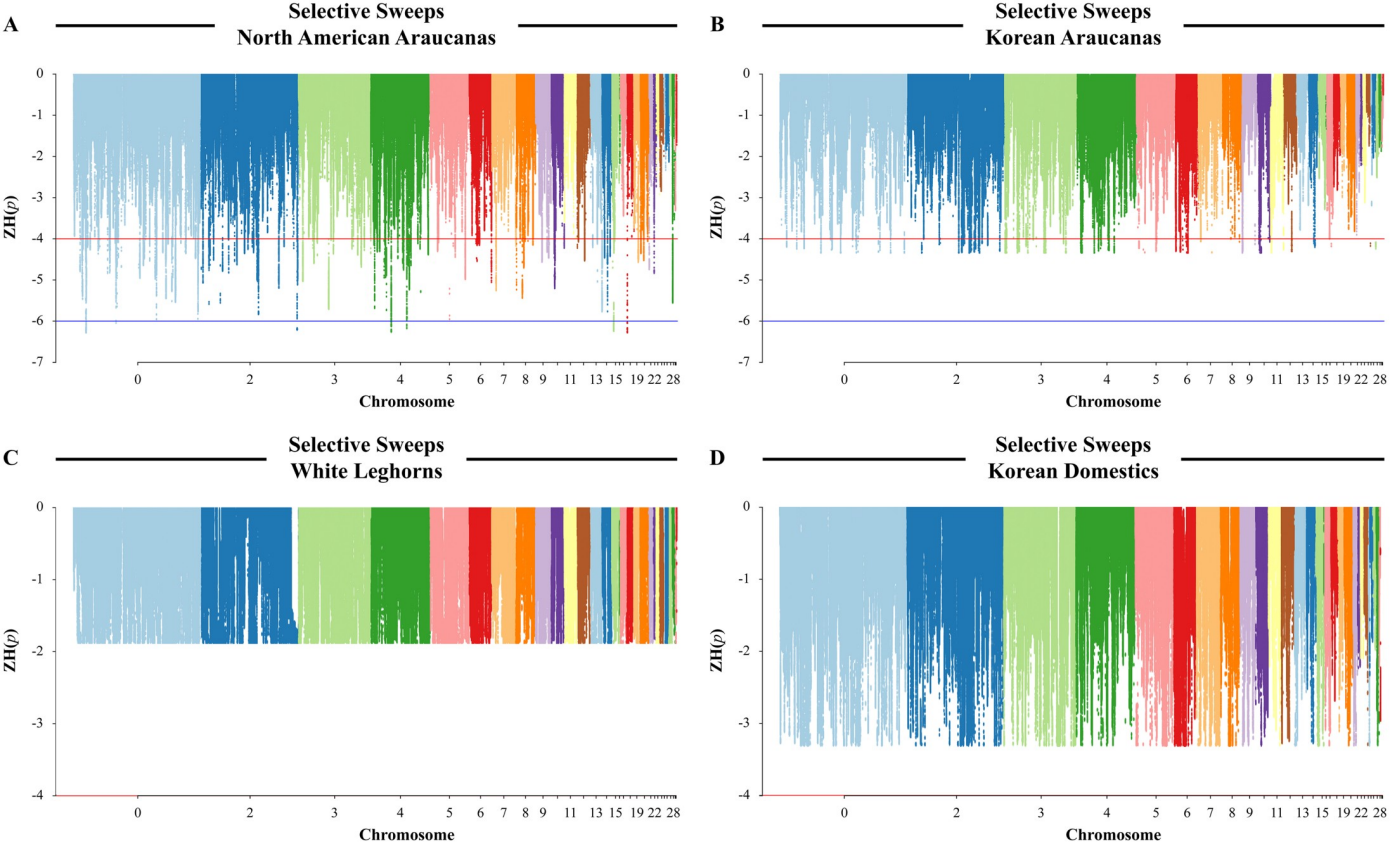
Selective sweeps. The negative tail of the ZH_*p*_ distribution presented along chromosomes 1–28, 30, 32, 33, and LGE64. The Z and W chromosomes were not included because the sex of many birds in the study was not reported. Each dot represents a creeping window of up to 40 kb. The horizontal red line stands for the suggestive sweep significance level at ZH_p_ = -4. The horizontal blue line stands for the strong sweep significance level at ZH_p_ = -6. (A) Results from six North American Araucana, (B) results from three Korean Araucana, (C) results from three White Leghorns, and (D) results from three Korean Domestics.

Another method for identifying genes associated with the NAA breed is to look for genomic regions where selection is occurring within the genome. A creeping window method was used to visualize the distribution of pooled heterozygosity within each breed [19]. A total of 9,422,906 SNPs were used in the selective sweep analyses. The negative ZH_p_ distribution plot showed several regions with ZH_p_ ≤ -4 in NAA and KA, indicating suggestive selection (Fig 4A and 4B). The NAA also had regions with ZH_p_ ≤ -6, indicating strong selection. The KA, being more recently derived from a NAA (a Golden Duckwing Araucana) and WL [15], had ZH_p_ values as low as -4.33, which indicate only the presence of moderate selection. One explanation for the discrepancy in the range of ZH_p_ values between the two groups is the difference in the number of individuals; the NAA group has six chickens, whereas the KA group has only three chickens. The ZH_p_ calculation is influenced by the number of reads present and having twice as many birds increase the number of SNPs used in the calculation [19,20]. To overcome any bias the number of chickens may contribute, a representative subset of NAA the same frequency of NAA phenotypes as in the full set was selected. Selective sweeps based on this half set of NAA, equal to the number of KA birds, were recalculated. All of the strong selective sweeps identified in the full NAA dataset passed the suggestive selective sweep threshold in the half NAA dataset, with -4.44 ≥ ZH_p_ ≥ -4.8. Comprehensive descriptive statistics of the negative ZH_p_ values for the KA and half set of NAA are provided in Table 3.

**Table 3:** Descriptive statistics of the negative ZH_p_ values for all conducted selective sweeps.

Statistic	NAA (n = 6)	NAA (n = 3)	KA	KD	WL
Maximum -ZH_p_	0	0	0	0	0
Minimum -ZH_p_	-6.27	-4.8	-4.33	-3.31	-1.87
Mode (NModes)	0 (20,831)	0 (20,283)	-0.02 (17,509)	-3.31 (31,397)	-1.87 (171,172)
Median	-0.59	-0.62	-0.69	-0.68	-0.80
Mean	-0.86	-0.90	-0.89	-0.94	-0.93
Standard deviation	0.87	0.86	0.79	0.86	0.63
# of Creeping windows	1,885,372	1,839,836	1,926,092	1,789,416	1,993,801

Based on these data, selective sweeps in the full set of NAA were identified on chromosomes 1–10, 12–15, 18–22, and 28. In the KA, selective sweeps were identified on chromosomes 1–12, 14, 17, 24, and 27. The negative ZH_p_ values for KD and WL were all greater than -4.0, indicating the lack of any significant selective sweeps based on this dataset (Fig 4C and 4D).

There were 18,758 creeping windows in NAA that reach the suggestive selection threshold of -4.0 ZH_p_ on 20 chromosomes (1–10, 12–15, 18–22, and 28) and of those 949 were at the strong selection threshold of -6.0 ZH_p_ (S4 Table). After combining overlapping windows above the strong selection threshold, there were a total of eight strong selective sweeps on five chromosomes (1, 2, 4, 15, and 18) in NAA (Table 4). These genetic regions have significantly reduced levels of heterogeneity and contain 15 genes, listed in Table 4.

**Table 4:** Regions defined as strong selective sweeps in six NAAs.

			Full NAA set (6 birds)			Half NAA set (3 birds)			KA set (3 birds)			
chr	start	stop	windows	Avg ZH_p_	SD	windows	Avg ZH_p_	SD	windows	Avg ZH_p_	SD	Known gene(s)
1	19,176,800	19,225,898	10	-6.19	0.11	10	-4.72	0.10	10	1.05	0.12	None
1	19,248,994	19,452,814	376	-6.23	0.04	376	-4.77	0.04	376	0.87	0.70	None
1	65,229,480	65,268,953	1	-6.04	-	1	-4.56	-	1	1.99	-	*SLCO1B3*, ENSGALG00000019276
2	147,275,921	147,337,002	33	-6.19	0.01	33	-4.69	0.02	33	0.33	0.15	*TSNARE1*
4	31,400,689	31,482,936	68	-6.11	0.06	68	-4.78	0.01	68	-0.96	0.12	*SMAD1*, *MMAA*, ENSGALG00000043141, ENSGALG00000028322, *ZNF827*
4	55,307,353	55,357,189	4	-6.13	0.04	4	-4.65	0.05	4	0.64	0.06	*SEC24D*, *METTL14*
15	2,976,357	3,132,521	389	-6.14	0.07	427*	-4.69	0.08	427	0.91	0.26	*STX2*, *RF02271*, *ADGRD1*
18	390,709	469,520	68	-6.19	0.07	68	-4.71	0.07	68	1.31	0.10	*MYH1D*, *MYH1F*
		Total	949			987			987			

*Half group windows overlap with full group, no windows outside of full group were counted.

Selective sweeps in KA consisted of 17,427 creeping windows that reach suggestive selection (ZH_p_ ≤ -4) on 16 chromosomes (1–12, 14, 17, 24, and 27) (S5 Table). Violin plots were created for each group from all of their negative ZH_p_ values to visually show the differences in distribution (S2 Fig). Comparative genomics showed that levels of homogeneity within the individual chicken breeds are generally low, whereas levels between the breeds are highly variable.

### North American Araucana traits

RF analysis were able to identify 203 markers to classify NAA from the other three groups. The selective sweep analysis identified the presence of 8 strong selective sweeps in NAA, and only suggestive sweeps in the KA. Prior to combining the candidate gene sets from both analyses to identify genes not previously known to be associated with NAA phenotypes, the results were interrogated to confirm the presence of significant markers and sweeps containing or nearby the four previously identified genes/regions associated with characteristic traits of NAA: blue eggshell, ear-tufts, a pea comb and rumplessness.

The breed standard for NAA requires a blue-colored eggshell [10]. The gene product of *SLCO1B3* is a membrane transporter of amphipathic organic compounds, including bile salts. The blue eggshell color is associated with ectopic expression of *SLCO1B3* in the shell glands of the uterus in birds with an *EAV-HP* insertion in the 5’ flanking region of the gene (chr1: 65,220,675), and is thought to be responsible for blue eggshells in Araucana chickens [21]. Direct analysis of each NAA genome has identified the *EAV-HP* insertion in all six NAA birds. It was further confirmed that the three WL birds lacked the insertion [16]. Whereas there are markers within the region surrounding and encompassing *SLCO1B3*, RF did not select any of these markers as significant classifiers in either analysis. This is likely due to the presence of blue eggshell laying KA, which posses the same insertion in the same position as NAA [15]. In NAA, there exists one creeping window which passes the threshold for a strong sweep starting between *SLCO1B3’s* 4^th^ and 5^th^ exons and continuing beyond its sequence and into an adjacent novel gene (ENSGALG00000019276), but this occurs after the site of the *EAV-HP* insertion. There is also a suggestive selective sweep starting upstream of *SLCO1B3*, before the *EAV-HP* insertion site, and stretching to include the strong selective sweep creeping window and beyond it. We hypothesize that the suggestive selective sweep in conjunction with the strong selective sweep creeping window supports the selection of blue eggshell color in NAA. In the KA, the suggestive selective sweeps on chromosome 1 did not overlap the region upstream or including *SLCO1B3*.

Another breed standard in NAA are ear-tufts [10]. These are formed by feathers protruding from fleshy peduncles found in the ventral region of the external ear canal. Two genes, *TBX1* and a partial sequence of *GNB1L* are heterozygous for ear-tufted NAA birds [13]. The representation of markers in this region was low. A total of 354 markers passed filtering, with only one in *TBX1*, 334 in *GNB1L* and 19 intergenic. Whereas there are some SNP markers that passed filtering within this region, none of them were significant in either RF or selective sweep analyses. This may be due to low coverage within this region and the inclusion of two clean-faced birds, making the NAA population heterogeneous. All of the KA, KD, and WL are clean-faced, and no sweeps exist on microchromosome 15 in KA. SvABA was used to examine the chromosome for structural variations. Twenty-one deletion breakpoints were identified on 15 in NAA, see Table 5. Of these deletions (88–8,389 bps long), seven were present in one or more ear-tufted birds and were absent in the clean-faced NAA. Deletions in three of these regions were also completely absent from the KA, KD, and WL birds. Only the longest deletion was found to be heterozygous in all four ear-tufted birds, while absent in the other 11 birds. This deleted region (15:1,019,583–1,027,972) includes an entire exon and neighboring intron sequence within gene *TXNRD2* (thioredoxin reductase 2). This heterozygous deletion falls within the heterozygous region previously identified by genome-wide association study as associated with ear-tufts [13]. A homologous region is deleted in most cases of DiGeorge syndrome, and haplo-insufficiency of *TXNRD2* has been considered for contributing to the phenotype for DiGeorge syndrome [22,23].

**Table 5:** Break end structural variants present in study population on chromosome 15 (NC 006102.4). All tufted birds are characterized by an 8.3kb heterozygous deletion on chromosome 15.

			North American Araucanas														
			Tufted		Tufted		Clean		Tufted		Clean		Tufted				
START	STOP	SIZE	NAA01		NAA02		NAA04		NAA04		NAA05		NAA06		Gene	Description	Position
347,555	348,072	517	U	(0/1)	✓	(1/1)	NA	(0/0)	NA	(0/0)	NA	(0/0)	✓	(1/1)	*UBE2L3/AL158801*.*1*	ubiquitin conjugating enzyme E2 L3	intronic
1,019,583	1,027,972	8,389	✓	(0/1)	✓	(0/1)	NA	(0/0)	U	(0/1)	NA	(0/0)	U	(0/1)	*TXNRD2*	thioredoxin reductase 2	exon (whole)
2,166,472	2,167,057	585	✓	(1/1)	✓	(0/1)	NA	(0/0)	✓	(1/1)	✓	(1/1)	✓	(1/1)	No genes	NA	intergenic
2,826,383	2,826,602	219	✓	(0/1)	U	(1/1)	✓	(1/1)	✓	(1/1)	NA	(0/0)	✓	(1/1)	*SFSWAP*	splicing factor SWAP	intronic
3,225,855	3,226,300	445	✓	(1/1)	NA	(0/0)	NA	(0/0)	NA	(0/0)	NA	(0/0)	NA	(0/0)	*RIMBP2/RBP2*	RIMS binding protein 2	intronic
4,106,455	4,106,572	117	✓	(0/1)	NA	(0/0)	NA	(0/0)	NA	(0/0)	NA	(0/0)	U	(0/1)	*LOC107051578*	uncharacterized (long non-coding RNA)	intronic
4,189,586	4,189,756	170	✓	(0/1)	NA	(0/0)	NA	(0/0)	NA	(0/0)	NA	(0/0)	NA	(0/0)	No genes	NA	intergenic
4,264,865	4,270,692	5,827	U	(1/1)	U	(0/1)	U	(0/1)	U	(1/1)	NA	(0/0)	✓	(1/1)	*LOC107051609*	uncharacterized (long non-coding RNA)	intronic
4,280,664	4,281,215	551	✓	(1/1)	U	(0/1)	✓	(1/1)	NA	(0/0)	✓	(1/1)	NA	(0/0)	*LOC107051609*	uncharacterized (long non-coding RNA)	intronic
5,075,785	5,076,236	451	✓	(1/1)	NA	(0/0)	NA	(0/0)	NA	(0/0)	NA	(0/0)	✓	(1/1)	*LOC107051605*	uncharacterized (long non-coding RNA)	exon (partial)
5,218,248	5,218,471	223	NA	(0/0)	NA	(0/0)	NA	(0/0)	NA	(0/0)	✓	(0/1)	✓	(0/1)	*PITPNM2*	phosphatidylinositol transfer protein membrane associated 2	intronic
6,597,976	6,598,893	917	NA	(0/0)	NA	(0/0)	NA	(0/0)	NA	(0/0)	✓	(0/1)	✓	(1/1)	*CORO1C*	coronin 1C	intronic
7,598,349	7,598,465	116	NA	(0/0)	✓	(0/1)	✓	(0/1)	NA	(0/0)	NA	(0/0)	NA	(0/0)	*TTC28*	tetratricopeptide repeat domain 28	exon (partial)
7,720,778	7,720,969	191	NA	(0/0)	NA	(0/0)	NA	(0/0)	NA	(0/0)	✓	(0/1)	NA	(0/0)	*TTC28*	tetratricopeptide repeat domain 28	intronic
7,746,729	7,747,057	328	✓	(0/1)	NA	(0/0)	NA	(0/0)	NA	(0/0)	✓	(0/1)	NA	(0/0)	*SLC2A11L5* (*LOC416916*)	solute carrier family 2 member 11-like 5	intronic
7,838,965	7,839,053	88	✓	(1/1)	NA	(0/0)	NA	(0/0)	NA	(0/0)	U	(0/1)	NA	(0/0)	*ZNRF3*	zinc and ring finger 3	intronic
9,286,836	9,287,033	197	NA	(0/0)	✓	(1/1)	✓	(1/1)	✓	(1/1)	U	(1/1)	U	(1/1)	No genes	NA	intergenic
9,608,637	9,609,387	750	NA	(0/0)	U	(0/1)	NA	(0/0)	NA	(0/0)	✓	(0/1)	U	(0/1)	No genes	NA	intergenic
10,428,724	10,428,823	99	NA	(0/0)	✓	(1/1)	NA	(0/0)	NA	(0/0)	NA	(0/0)	NA	(0/0)	*RTN4R*	reticulon 4 receptor	intronic
10,746,849	10,747,291	442	✓	(1/1)	U	(1/1)	✓	(1/1)	NA	(0/0)	✓	(1/1)	✓	(1/1)	*LOC107054685*	uncharacterized (long non-coding RNA)	intronic
12,123,689	12,123,786	97	✓	(1/1)	NA	(0/0)	✓	(1/1)	NA	(0/0)	NA	(0/0)	NA	(0/0)	*LOC107054674*	uncharacterized (long non-coding RNA)	intronic

NA—Structural variant not present in vcf

U—Structural variant present in unfiltered vcf

✓—Structural variant present in filtered vcf (passed filtering)

0/1—Heterozygous for structural variation

1/1—Homozygous for structural variation

0/0—Homozygous for reference alignment

The pea comb is a required feature for breed recognition in NAA [10]. A causative mutation for a pea comb was identified as a duplication in the first intron of *SOX5* [24], which is on chromosome 1 with ~3,102 SNPs within this region of the gene. Based on the filtered SNP dataset, markers in this region were not found to be significant in either RF or selective sweeps analyses. CNV-seq was used to detect any copy number variation on chromosome 1 in birds with a pea comb. All six NAA and three KA, with median log_2_ ratios of 2.08–3.81, possess the chromosome 1 duplication in the first intron of *SOX5* (5–6 creeping windows between 65,888,001 and 65,902,000 bp) when compared to the WL (Table 6). The KD showed no difference in copy number variation associated with a pea comb when compared to the WL. These results confirm that NAA and KA have the pea comb phenotype; whereas KD and WL have regular-sized combs and wattles. Based on the median log_2_ ratios, there appears to be greater variation in the size of the duplication in the NAA, which may be due to the older age of the breed, giving it time to accrue more variation in this region.

**Table 6:** Detecting the mutation for pea comb by identifying copy-number variations in *SOX5*. This table contains the median value of Log_2_ ratios comparing WL to NAA, KA and KD in the first intron of *SOX5*.

	WL01	WL02	WL03
**NAA01**	2.34	2.31	2.38
**NAA02**	3.73	3.81	3.78
**NAA03**	2.26	2.34	2.25
**NAA04**	2.08	2.11	2.19
**NAA05**	3.30	3.35	3.32
**NAA06**	3.09	3.18	3.15
**KA01**	2.85	2.93	2.70
**KA02**	2.94	3.02	2.99
**KA03**	2.94	3.02	3.00
**KD01**	0.05	0.09	0.17
**KD02**	-0.2	-0.11	-0.08
**KD03**	-0.04	0.01	0.08

Confirming our earlier finding, the two candidate causative SNPs for rumplessness (*Rp*) were identified proximal to *IRX1* and *IRX2* [14]. Both of these SNPs (chr 2: 86,770, 373, SV1 and 86,870,271, SV2) were present in the filtered SNP dataset and held true for the *Rp* mutation. The single tailed NAA and all KA, KD, and WL were wildtype *A/A* at the *Rp* loci, as expected. The five rumpless NAA were *A/C* or *C/C* at both *Rp* loci. Neither SNP position was found to be significant in classifying the NAA using RF, but this is explained by the presence of the single tailed NAA preventing the *Rp* SNPs from fitting a discriminant pattern, which the RF models are sensitive to. With selective sweeps, the second *Rp* locus is present in 11 creeping windows in NAA that pass the threshold for suggestive selection; however, the first locus is absent from any windows for NAA. We hypothesize that the suggestive selective sweep including the second *Rp* locus confirms that *Rp* has been selected for in NAA. Neither locus exists in any sweeps in the KA consistent with KA being a tailed breed not having the *Rp* genotype.

To further validate our findings, genotyping of several wildtype chicken breeds, tailed NAA, and partial and rumpless NAA birds were performed using DNA extracted from blood samples. Chickens retain the nucleus within their erythrocytes enabling genotyping of blood from a wing vein. Tailed birds of 16 breeds (42 birds) were all wildtype having *A/A* loci for both SNPs, as were all 18 tailed NAA birds (Table 7). A heterozygous *A/C* genotype was found in both SNPs in 19 partial tailed NAA and 26 rumpless NAA birds. The homozygous *C/C* genotype was found in both SNPs for 32 rumpless birds. In two cases, partially tailed birds were identified with a SV1 *A/C*:SV2 *C/C* genotype, and in three further cases, a SV1 *C/C*:SV2 *A/C* genotype was identified in rumpless NAA. Out of the total of 141 birds tested none were identified that carried an *A/C* or *C/C* genotype in either the SV1 or SV2 SNPs where the other SNP carried the wildtype *A/A* loci.

**Table 7:** Results from genotyping of wildtype, tailed, partial and rumpless NAA chicken breeds for SNP variants SV1 and SV2 from wing vein blood.

	total number of birds tested	SV1	one or both alelles	SV2	one or both alelles
**Tailed araucana**	18	18 *A/A*	both	18 *A/A*	both
**Tailed wildtype**	42	42 *A/A*	both	42 *A/A*	both
**Partial tail araucana**	21	19 *A/C*	one	19 *A/C*	one
		2 *A/C*	one	2 *C/C*	both
**Rumpless araucana**	61	26 *A/C*	one	26 *A/C*	one
		32 *C/C*	both	32 *C/C*	both
		3 *C/C*	both	3 *A/C*	one

### Annotation of SNPs within each breed

VEP analysis was conducted on the SNP dataset after removing markers within each group where all samples were homozygous for the reference allele. The NAA had the highest percentage of positions with the alternate allele present (81.28%), followed by the KA (68.84%), then the KD (62.02%), and finally the WL (52.02%) (S6 Table). The number of SNPs grouped based on their most severe consequence is described in S7 Table. NAA and KA had SNPs on chromosomes 1–28, 30, 32, 33, and LGE64. KD and WL had SNPs on chromosomes 1–28, 32, 33, and LGE64.

### Functional annotation of the genes associated with NAAs from RF and selective sweeps analyses

The 26 candidate genes associated with SNPs from RF analysis that classify NAA from the other three groups, and the 12 identified genes from the strong selective sweeps in NAA were combined, and GO terms for all genes were compiled. Seven novel genes lacking any annotation were identified and were therefore not included. GO terms were associated with each gene and their associated GO molecular function, and cellular component ontology is detailed in S8 Table. Additional analysis of biological significance was performed (S9 Table), revealing key roles for these genes, including: actin binding and cytoskeleton; positive regulation of cell-substrate adhesion and cell adhesion; cell cycle control, DNA repair and centrosome proteins; G-protein components (G-protein coupled receptors, GTPases and Guanine nucleotide exchange factors); a metabolism gene and a ubiquitin ligase; metal ion, nucleic acid and RNA binding genes; signal pathway and transduction genes and a transcription factor; transmembrane transport and several vesicle trafficking associated genes.

## Discussion

The North American Araucana (NAA) breed is the result of crossing the Chilean “Collonocas” breed that laid blue eggs and was rumpless and a second breed called the “Quetros” that had ear-tufts but was tailed and laid brown eggs [10,11]. Carbon dating analysis of chicken bone samples from Chile suggests that these birds may have been introduced to South America from Polynesia. However, comparison of mitochondrial DNA haplotypes of these same samples revealed that they cluster more closely with chickens of European descent. Further analyses of the NAA genome may be useful for determining the origins of its ancestral breeds [25–27].

Careful breeding of these birds has continued to select for now characteristic blue eggshell color, ear-tufts, a pea comb, and rumpless phenotypes recognized in the breed standard [10]. Over time, gene variants and genomic regions have become fixed and define these NAA-specific characteristics [28]. To identify further breed specific variants, six NAA birds were resequenced and compared to the Gyeongbuk/Korean Araucana (KA), Korean Domestic (KD) and White Leghorn (WL) breeds.

A 71-fold coverage of the NAA chicken genome was generated. The high coverage sequence data generated herein provides an additional resource for future studies in the NAA breed. The filtered marker set of 9,170,378 SNPs successfully showed clear separation of the NAA birds from the three other lines in this study, confirming the presence of two NAA genetic traits underlying characteristic phenotypes, and importantly, identified candidate genes and pathways suggesting potential additional unique variants. Within the markers, SNPs that separate and classify NAAs from the other three groups of birds were identified. Principal component analysis clusters NAA together, and they are more similar to members of this group than the other nine birds. The dendrogram, based on dissimilarity between individuals, confirms this finding. We suggest that SNPs within the dataset are within or adjacent to genes that are deterministic for additional NAA traits. To this end, RF and selective sweeps subset the SNP list and rank its members in order of significance to separation of these groups. The SNPs identified by RF do not appear to cause mutations in the gene coding regions; however, they link to putative regulatory regions of the nearby genes.

In contrast, genes identified by selective sweeps, encompass whole or significant portions of gene(s) in addition to the surrounding regulatory regions. Therefore, priority was given to characterizing genes identified by selective sweeps. Several genes identified for meat quality or muscle growth and development have been previously studied in chickens and may point to additional NAA characteristic traits [29–31]. Further study of these genes in NAAs may reveal an allele or regulatory function that could increase the quality, supply, and/or diversity of commercial flocks in the future. The remaining two NAA genetic traits were confirmed by interrogating the alignment files with copy number variation and structural variant discovery tools. The 8.4kb hemizygous deletion found on microchromosome 15 in ear-tufted NAAs contains a portion of the gene TXNRD2, and is adjacent to a region previously identified to be associated with ear-tufts in NAAs (8). A microdeletion in humans containing *TBX1* and surrounding genes, including TXNRD2, has been linked to DiGeorge syndrome (22q11.2 deletion syndrome), the most common microdeletion disorder in humans [22]. Human populations have high rates of heterogeneity, and thus require large numbers of individuals to map hereditary diseases [32]. Therefore, using a single breed of chickens, with a lower rate of heterozygosity, as a model to study a trait that has similarities to a human disease would be advantageous. Additional data are necessary to determine the significance of the structural variants and the selective sweeps identified herein.

Whole-genome resequencing of KA, developed by crossing a Golden Duckwing Araucana (NAA) and WL, a recently developed blue eggshell laying chicken breed, reveals its origin and genetic characteristics [15]. The Golden Duckwing Araucana has the characteristic rumpless and ear-tufted traits, a pea comb, as well as blue-shelled eggs. Crossing produced the KA that shares the blue eggshell color and the pea comb phenotype of NAA, but are clean-faced and tailed, indicating that not all of the defining characteristics of the breed standard have been successfully transferred during a crossing of the NAA and WL lines. This may benefit the overall fitness of the KA as an egg layer, as the homozygous ear-tufted trait is associated with a lethal effect, and the rumpless trait can give rise to a short-back phenotype that is undesirable. The blue eggshell color is a result of a 5’ *EAV-HP* insertion that promotes expression of the transmembrane protein *SLCO1B3* in the uterus. *EAV-HP* is an ancient retrovirus found in many loci in modern birds, but in this instance, it is thought that biliverdin deposition, which produces the blue eggshell color, is enhanced in animals with *SLCO1B3* expression in the uterus. The pea comb trait is caused by duplication in the first intron of the *SOX5* gene. The expansion of this region leads to ectopic expression of *SOX5*.

In conclusion, this comparative genomic analysis demonstrates that the NAA possesses the genetic variants that give rise to the characteristic phenotypic traits associated with the breed. Breeders have maintained variants for clean-faced, as well as tailed traits, which are advantageous for future genetic studies. Additionally, the screening and direct sequencing of 141 tailed and rumpless chickens demonstrates that two SNPs upstream of the *IRX1/2/4* cluster are likely causative for gain-of-function misexpression of *IRX1* and *IRX2* that result in the rumpless phenotype. Also of importance, direct analysis of the six NAA genomes identified a heterozygous deletion in only the four ear-tufted birds in a region significantly associated with the trait. This finding may help breeders understand why there appears to be a lethal effect in birds homozygous for the ear-tufts trait. The results in this study present the NAA as a unique breed with some still unconfirmed breed specific traits making future work appealing to breeders, the commercial poultry industry, and geneticists.

## Materials and methods

### Ethics statement

The use of chicken embryos up to E15 does not require IACUC approval. For embryos from E15—E21 and hatched birds, blood and tissue collection was approved by the Clemson University IACUC protocol number 2011–041. Processing of blood and tissue samples was approved by the Clemson University IBC protocol number 2010–041 and 2017–08.

### Study cohort/sample collection and genotyping

North American Araucana (NAA) samples were collected from geographically distinct flocks that had not been interbred. Members of the Araucana Club of America were contacted to solicit research participants. Whole blood was collected from the wing vein and DNA extracted using the DNeasy Blood and Tissue Kit (QIAGEN, Valencia, USA). NAA samples were separated into fully or partially rumpless and tailed Araucana based on the morphology of the spine, and recorded at the time the blood sample was taken together with photographic images of each bird from our previous study [13]. Among the birds selected for whole-genome re-sequencing were females and males, tailed and rumpless, clean-faced and ear-tufted. For the genotype validation studies DNA from the original GWAS were used [13], as well as DNA from the Clemson University Poultry Farm Araucana and wildtype flocks, and additional varieties of tailed birds from the University of Georgia Athens poultry collection. PCR was performed using forward and reverse primers for SV1 (forward: ATCCTTATGAACTCCACAGACCAAA reverse: AGAATGAATTGGTTTAGTATCATCCAGA) and SV2 (forward: TATTCATAGAGGAGAGGAAACAACC reverse: GTTGTTGAACTCAGTGATGTATCA). Amplicons were sequenced by Eton Biosciences.

### Whole-genome resequencing

Genomic DNA adjusted to a concentration of 50 ng/μL in 10 mM Tris-CL buffer pH 8.5, measured by optical density using a NanoDrop 1000 Spectrophotometer (Thermo Scientific, Wilmington, USA). One hundred fifty ng of DNA was used for 2x100 bp paired-end sequencing, on an Illumina HiSeq 2000 at The Roslin Institute at The University of Edinburgh (Easter Bush, Midlothian, Scotland, UK). The following fragments were gel size selected: 440–489 bp.

Six samples were pooled per lane, and 6 lanes of data were generated. Raw sequence data is available at the NCBI sequence read archive, BioProject PRJNA524911. Additionally, nine chicken samples from PRJNA291174 were included in this project’s meta-analyses. The reads from these samples were also 2x100 bp paired-end and sequenced on an Illumina HiSeq 2000. These nine samples consisted of three Gyeongbuk/Korean Araucana (KA), three Korean Domestics (KD), and three White Leghorns (WL).

### Data preparation

The quality of each set of reads was examined using FastQC 0.10.1 [33]. Trimming and sorting of all reads were conducted using Trimmomatic 0.30 [34]. The output of forward and reverse, paired and unpaired reads were re-examined to verify an improvement in quality. Some of the metrics used to assess quality include (a) improvement in the per base sequence quality, (b) per sequence quality scores, (c) absence of overrepresented sequences, and (d) low kmer content [34]. Paired-end reads were trimmed for low quality scores in the leading three bp, trailing six bp, and within a sliding window of size four bp. Reads were eliminated if their minimum length fell below 36 bp.

### Sequence alignment, variant discovery, and coverage

The assembly sequence for *Gallus gallus* reference (GCF 000002315.4) was downloaded from NCBI [35,36] and formatted for indexing in Bowtie2 2.3.1 [37]. Bowtie2 was used to align all 15 samples to the reference genome. SAMtools 1.6 [38,39] was used to convert the Bowtie2 output alignments for sorting and indexing. Picard tools 2.7.0 [40] was used to add read groups. Coverages were calculated at this step in the pipeline.

GATK 3.8–0 [41] was used for initial variant calling, realignment, and recalibration of the generated bam files using the recommended GATK Best Practices [42,43]. The Integrated Genome Browser was used to visualize results [44]. Final variant calling was handled by SAMtools 0.1.19 [37,39] and BCFtools [37,39] resulting in a merged VCF with all samples. Filtering of the variants was handled by SnpSift 4.3t [45], vcftools 0.1.15 [46], and vcflib 1.0.0-rc1 [47] for SNPs only, mapq > 40, qual > 40, read depth ≥ 3, 0% missing data, positions that were biallelic, and autosomes. The resulting merged VCF was used for SNPRelate and random forest (RF) analyses. Vcftool was then used to subset each group of samples into separate files for use in the selective sweeps and Ensembl’s variant effect predictor [48] analyses.

### Identifying SNPs and regions associated with NAAs

To compare the variation present between the four groups of samples, the previously filtered and merged VCF file was used as input to Bioconductor’s SNPRelate package [49] in R 3.5.0 (R Core Team, 2013). The VCF was converted to a genofile, and a principal components analysis plot and a dendrogram were produced to show the relatedness of all 15 samples, using default parameters. The dendrogram is based on the dissimilarity that exists between each pair of samples.

The genofile from SNPRelate was used as input for discriminant analysis using RF. RF was run in the R package randomForest [50] using the standard command randomForest. The MDS plot from RF analysis was plotted in Matlab using custom scripts and polished in Adobe Illustrator (Adobe, Inc.). The importance scores were imported from RF analysis and sorted for Mean Decrease in Accuracy to determine top contributing SNPs for group separation. The top 22 SNPs from the sorted list that were located within or adjacent (upstream or downstream) to a gene, had at least one copy of the alternate allele present in a majority of NAA, and fit the pattern of being heterozygous or fixed for the alternate allele in NAA and heterozygous (opposite of NAA) or homozygous reference allele in KA, KD, and WL were further examined for genes that could be associated to NAA specific traits. Performance of the model was assessed based on cluster separation in MDS plot using the Monte Carlo permutation procedure of the Davies-Bouldin Index.

The top 22 SNPs were all non-coding variants within a gene, and in order to address the possibility that these might be regulatory variants, a modified version of the galGal5 GFF file, containing only the entries for full genes, the fourth column for each row contained “gene”, and bedtools closest were used to identify the nearest ten genes to each marker [51]. The gene list was then filtered by distance from marker, keeping only those genes within 40kb upstream (negative value) or downstream (positive value). This distance was chosen as it is recognized as where linkage disequilibrium breaks down in chickens [18].

To identify selective sweeps, the creeping window method described by Qanbari and coworkers was implemented [19]. VCF files that were merged by group with 9,422,906 SNPs were used as input. Parameters included a window size of no larger than 40 kb, gaps of less than 10 kb between SNPs, and a minimum of 50 SNPs per window. Resulting negative ZH_p_ values were plotted in R using modified code from the qqman package [52]. The manhattan plot code was modified to plot the negative ZH_p_ values instead of -log_10_(*p*) values and the suggestive and genome-wide lines were moved to suggestive and strong sweep cutoffs. All creeping windows with a ZH_p_ ≤ -6 were considered to be under strong selection and were reviewed for the presence of genes and SNPs. Creeping windows with a ZH_p_ ≤ -4 were considered to be under suggestive selection and were checked for the presence of known genes/markers for North American Araucana phenotypic traits (blue eggs, a pea comb, rumpless, and tufted) [14,21,24].

### Annotation with gene ontology (GO) term analysis and pathways

All putatively identified genes from RF and selective sweeps analyses were annotated with their GO terms, and sorted for the most represented terms. The top 10 most common terms were reviewed, and their associated genes were analyzed for relevance to NAA phenotypes. Pathway information was initially collected from KEGG GENES [53–55], and Ensembl [56] was used as a secondary source for any genes missing annotation.

### Predicting consequences of alternate alleles in NAAs

Vcftools was used to remove all positions within each group where all individuals were homozygous for the reference allele. Then the VCF file for each group was processed by Ensembl’s VEP script release 93.0 [48] with database 93 Gallus gallus 5.0 to assess and categorize the location and/or type of consequence predicted for each SNP. SNPs were characterized based on location (intergenic, intronic, upstream, downstream, splice site, 5’ UTR, or 3’ UTR) or type of change (frameshift, missense, synonymous, stop gained, stop lost, stop retained, or within mature miRNA).

For a secondary method to predict the possible implications of the SNPs identified as classifying for NAA by RF, snpEff 4.3t [45] was used to annotate the same NAA vcf file that was used for VEP. The input genome used by snpEff was Gallus gallus-5.0.86. Only the results for the 22 RF markers were extracted and combined those from VEP.

### Structural and copy number variations

The presence of structural variations in the 15 birds was determined using SvABA FH version: 134 [57]. The ‘assemble all reads’ option was used on the bam files with the Galgal5 reference. The unfiltered and filtered structural variants vcf files were filtered for results on chromosome 15 (NC 006102.4). All regions that passed filtering in at least one NAA were analyzed in both files for all other birds.

To get valid copy number variation results, the trimmed fastq files were all downsampled to the sample with the fewest reads using the sample command with seed 100 for seqtk 1.3-r106 [58]. Then, the same sequence alignment method was followed. Samtools was used to extract the list of best mapping locations from the downsampled bam files as described in the CNV-seq 2014/08/12 documentation [59]. Only the best hits for chromosome 1 were used as input to CNV-seq. The other command inputs were genome-size equal to the length of chromosome 1, window-size of 4kb, and annotate. Three sets of pairwise comparisons were made: 1) each NAA vs. each WL, 2) each KA vs. each WL, and 3) each KD vs. each WL. Variations were found to be significant with a p-value **≤** 0.001 and a log_2_ value **≥** 0.6 for a minimum of four overlapping windows. As stated in Wright, et al. 2009, the critical region containing the duplication for a pea comb is upstream of the first annotated exon in chickens, and their 5’ RACE analysis in the chicken showed the presence of the upstream exon 1 in chickens showing 90% identity to human *SOX5* exon 1. Using BLAT [60] on their published SOX-Duplication sequencing primers and the “BestRefSeq” annotation for the full gene and start of chicken exon 1 (proposed exon 2), a region to analyze for sequence duplications on chromosome 1:65,660,324–65,938,356 in the galGal5 reference was identified. The median of log2 ratios for each set of pairwise comparisons was calculated for a subset of the region (65,888,001–65,902,000bp).

Code and commands to perform all analyses within this manuscript are available in the S1 File.

## Supporting information

S1 Fig Flowchart of two methods used to identify candidate genes associated with North American Araucanas (NAAs). (PPTX)

S2 Fig Violin plot visualization of the selective sweep zHP frequency distribution for NAA, KA, KD and WL breeds. (TIF)

S1 Table Whole-genome resequencing data for 15 chicken samples. (XLSX)

S2 Table Results of random forest analysis to separate North American Araucanas from the three other groups. (XLSX)

S3 Table List of genes local to the 22 non-coding SNPs identified by random forest (RF). The SNPs identified by RF may harbor regulatory elements that affect nearby genes. Listed all genes within 40kb upstream (negative distance from gene) or downstream (positive distance from gene) of the 22 identified SNPs.
(XLSX)

S4 Table Selective sweep results for North American Araucanas. (XLSX)

S5 Table Selective sweep results for Korean Araucanas. (XLSX)

S6 Table Number of positions in NAA, KA, KD and WL where the alternate allele is present in at least one bird within the group. (XLSX)

S7 Table Number of SNPs grouped based on the most severe consequences. (XLSX)

S8 Table GO terms including molecular function and cellular components for candidate genes. (XLSX)

S9 Table Description and GO annotation for the genes associated with SNPs from RF and selective sweep analyses. (XLSX)

S1 File Command lines and scripts used to generate results. (TXT)
