# Supplementary material for: Discovery of genomic variations by whole-genome resequencing of the North American Araucana chicken
Source: PLoS One. 2019 Dec 10;14(12):e0225834. doi: 10.1371/journal.pone.0225834 (PMC6903725; doi:10.1371/journal.pone.0225834)
Supplement: S1 Fig — (PPTX) [file pone.0225834.s001.pptx]

## Slide 1
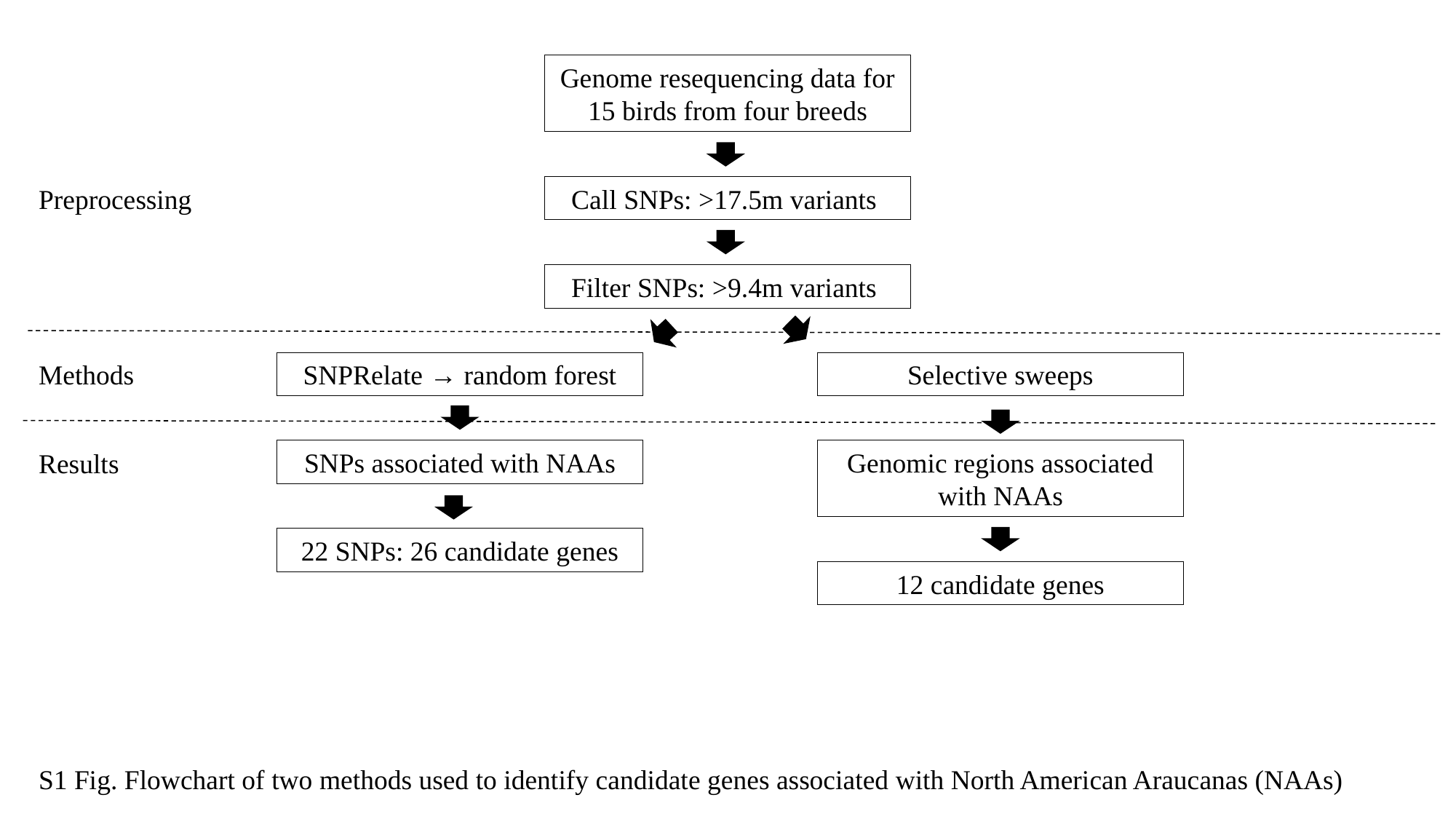

Genome resequencing data for 15 birds from four breeds
Preprocessing
Call SNPs: >17.5m variants
Filter SNPs: >9.4m variants
Methods
SNPRelate → random forest
Selective sweeps
SNPs associated with NAAs
Genomic regions associated with NAAs
Results
22 SNPs: 26 candidate genes
12 candidate genes
S1 Fig. Flowchart of two methods used to identify candidate genes associated with North American Araucanas (NAAs)
