# Supplementary figures and images for: Discovery of genomic variations by whole-genome resequencing of the North American Araucana chicken
Source: PLoS One. 2019 Dec 10;14(12):e0225834. doi: 10.1371/journal.pone.0225834 (PMC6903725; doi:10.1371/journal.pone.0225834)

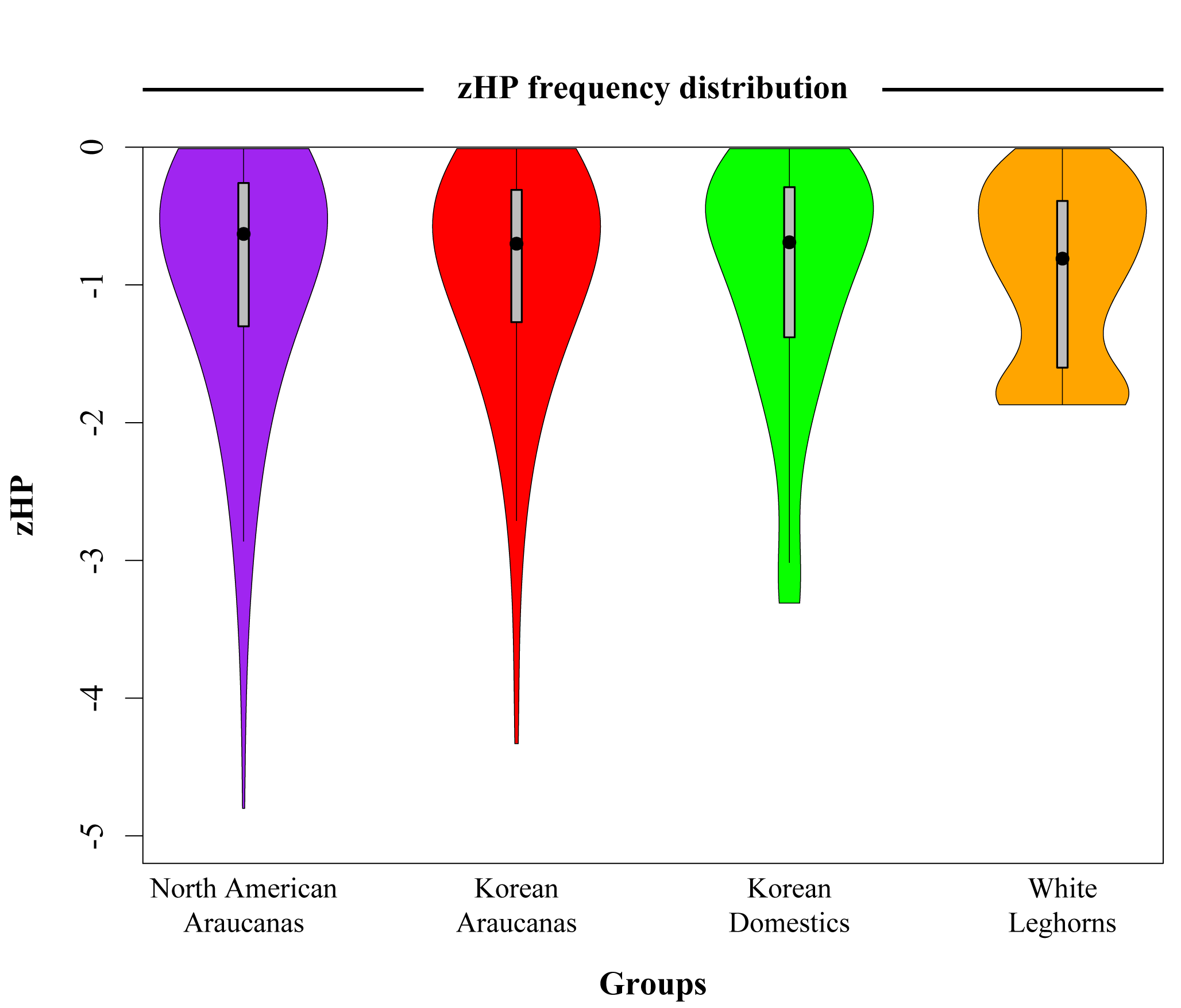

Supplement: S2 Fig — (TIF) [file pone.0225834.s002.tif]
